# Testing minimally invasive imaging methods for phenotypic sex determination in immature frogs

**DOI:** 10.3389/fvets.2026.1934243

**Published:** 2026-10-14

**Authors:** Nikolett Ujhegyi, Veronika Bókony, Árisz Ziszisz, Noémi Kovács, Ildikó Horváth, Maya Zsófia Rozsnyik, Richárd Limboczki, Ákos Benedek Lini, Gábor Berkei, Edina Nemesházi, Andrea Kásler, Zsanett Mikó, Domokos Máthé, Krisztián Szigeti, Emese Balogh

**Affiliations:** 1 Department of Evolutionary Ecology, Plant Protection Institute, HUN-REN Centre for Agricultural Research, Budapest, Hungary; 2 Department of Wildlife Biology and Management, Hungarian University of Agriculture and Life Sciences, Gödöllõ, Hungary; 3 Department of Exotic Animal-, Wildlife-, Fish- and Honeybee Medicine, University of Veterinary Medicine Budapest, Budapest, Hungary; 4 Department of Biophysics and Radiation Biology, Semmelweis University, Budapest, Hungary; 5 In vivo Imaging Advanced Core Facility, Hungarian Centre of Excellence for Molecular Medicine, Szeged, Hungary; 6 Csipet Csapat Veterinary Center, Budapest, Hungary; 7 Helvetic Clinics, Budapest, Hungary; 8 Konrad Lorenz Institute of Ethology, Department of Interdisciplinary Life Sciences, University of Veterinary Medicine Vienna, Vienna, Austria; 9 Department of Systematic Zoology and Ecology, ELTE Eötvös Loránd University, Budapest, Hungary; 10 Department of Zoology, University of Veterinary Medicine Budapest, Budapest, Hungary

**Keywords:** amphibians, computed tomography, gonad examination, juveniles, magnetic resonance (MR), *Rana dalmatina*, sexual maturity age, ultrasonography

## Abstract

Amphibians are regularly encountered in exotic veterinary practice, and knowledge of their sex can be extremely important for various programs for breeding and species conservation, as well as for veterinary care. However, sex identification remains challenging because many amphibians lack secondary sexual characteristics until reaching maturity, and genetic sex may not always correspond to phenotypic sex. Consequently, visual inspection of the gonads is often the most reliable method for identifying sex in amphibians. However, non-invasive imaging methods for gonad examination have not been formally validated in juvenile amphibians, where the small size of the gonads may limit visualization success. In our study, we attempted to determine the phenotypic sex of small-sized, immature and mature agile frogs (*Rana dalmatina*) using three non-invasive imaging methods: general veterinary ultrasound, native computed tomography (CT), and magnetic resonance imaging (MRI). Ovaries in immature females were not unambiguously recognizable with any of the tested methods, but became clearly visible during oocyte development, enabling identification in mature females with MRI and ultrasonography. Testes were identifiable in both immature and mature males by MRI but not by ultrasonography or CT. Weighing the costs and benefits of each method in light of our results, we make recommendations for further research into non-invasive sexing of juvenile amphibians.

## 1 Introduction

Amphibians are regularly encountered in exotic veterinary medicine as pets, zoo animals, and research subjects. These species play a critical role in their ecosystems and are often used worldwide as environmental health indicators in their habitats; yet, more than 2,000 amphibian species are listed as endangered, with vulnerable populations declining worldwide (1). Amphibians are one of the most interesting groups of vertebrates in terms of the diversity of their reproductive modes (1). For these reasons, amphibians are often involved in various kinds of breeding programs and research projects where knowing the sex of individual animals is important (2–7). Determination of phenotypic sex of sexually mature amphibians is generally based on primary or secondary sexual characteristics (2, 6, 8). The latter include sex-specific morphology (e.g., skin coloration, nuptial pads on the thumbs of males, body length and weight, or the size/swelling of the cloaca), behavior, or hormone profiles found in blood plasma, urine, or feces (3, 9). However, even in adults, these methods may be unreliable for sex identification. For hormonal measurements, a single test cannot provide 100% accurate results, as seasonal fluctuations, possible exogenous hormones, stress, or housing conditions can alter hormone profiles (6, 8, 10–15). External morphological signs of sexual dimorphism are often only visible in sexually mature individuals around the breeding season; the lack of environmental stimuli in captivity can often lead to misleading results (2, 5, 16, 17). Body size may differ statistically between the two sexes, but the ranges overlap significantly, so it only provides ~70%−75% accuracy even in adult frogs (6, 9). Alternatively, genotypic sex can be identified by molecular methods from minimally invasive samples such as blood or buccal swabs, although this approach has two drawbacks. First, while validated molecular markers for reliable sex identification are available for mammals (18) and birds (19), they are unavailable for most species of cold-blooded vertebrates. Most amphibians do not have distinct heteromorphic sex chromosomes, and polyploidy/aneuploidy is common, so molecular sexing requires species-dependent marker development (20–23). Second, genotypic sex can be overridden by environmental effects experienced during embryonic or larval development; thus, phenotypic sex is not always in concordance with genotypic sex (24, 25).

Therefore, direct examination of the gonads may be the only way of identifying phenotypic sex in many amphibians. However, this can be difficult to achieve without harming the animals, especially in small-sized species. Coelioscopy is a reliable method for direct gonad examination in amphibians (26), but it entails financial costs, health risks, and ethical concerns. As less invasive alternatives, various imaging techniques may be promising; yet, these have been explored in a relatively small number of amphibian species, focusing on sexually mature animals. To date, ultrasonography has been used successfully in adult animals—primarily females—to obtain useful information about reproductive status and can be utilized in breeding programs (3, 16, 27). For example, ultrasonography permits the diagnosis of presence or absence of mature eggs and whether oviposition was associated with complete or partial release of developed eggs (3, 26). Similarly, a few studies were also done with computed tomography (CT) and magnetic resonance imaging (MRI), finding these techniques suitable for identifying reproductive organs in adult females (9, 16, 28). In two frog species, adult males were also identifiable by both ultrasonography and MRI (16). As a further minimally invasive imaging technique, thermography has been successfully used in mammals to assess the condition of the reproductive organs (29, 30); however, the utility of this method for sexing amphibians is dubious given that their gonads are situated deep within the coelomic cavity whereas their skin temperatures are strongly affected by environmental conditions, by evaporative heat loss from the wet skin, and even by human handling (31, 32).

Determining the sex of juvenile individuals can be particularly useful for ensuring that animals of the appropriate sex are included in breeding programs at the right time. Yet, to our knowledge, no study has been done to evaluate whether widely available, non-invasive imaging techniques may be suitable for sex determination in immature amphibians, except for a few poorly reported cases in non-peer-reviewed literature (17, 33). As a first step toward this goal, we examined whether it is possible to identify the gonads in a small sample of sexually immature, small-sized frogs using minimally invasive imaging techniques, ranging from relatively easily accessible to highly specialized methods. Specifically, we investigated agile frogs (*Rana dalmatina*) at 1.8 and 2.5 years of age, raised in captivity for a long-term research program. In this species, sexual maturity is usually reached around 2 and 3 years of age in males and females, respectively (34, 35). To explore the feasibility of minimally invasive gonad imaging, we used ultrasound, computed tomography, and magnetic resonance imaging.

## 2 Materials and methods

### 2.1 Animals

This study was approved by the Ethics Committee of the Plant Protection Institute and licensed by the Environment Protection and Nature Conservation Department of the Pest County Bureau of the Hungarian Government (PE/EA/295–7/2018, PE-06/KTF/07949–6/2023, PE-06/KTF/00754–8/2022, PE-06/KTF/00754–9/2022, PE/EA/01171-6/2025). We investigated a total of 19 healthy agile frog individuals that were collected as freshly laid eggs from a wild population on 15 March 2023 and raised in captivity for research purposes (36) until 2025. We included eight males and 11 females in the imaging procedures reported here; the time schedule is shown in Supplementary Figure S1. During all imaging processes, the examiners were blind to the sex of the studied animals. We started the gonad examinations in April, when the animals had a mean body weight of 6.74 g (range: 3.39–11.16 g) and a mean snout-vent length (SVL) of 40.68 mm (min–max: 33.95–49.01 mm). At this time, none of the examined individuals had nuptial pads (which would indicate sexual maturity in males) or big bellies (which would indicate sexual maturity in females). We repeated selected imaging examinations in October–November as detailed below, when the animals had a mean body weight of 11.42 g (range: 5.82–17.99 g) and mean SVL of 45.46 mm (min–max: 35.2–51.5 mm). At this time, secondary sexual characteristics were already apparent in some individuals, indicating sexual maturity. All individuals' phenotypic (gonad) sex was identified by coelioscopy in October following established protocol including proper analgesia (26).

### 2.2 Anesthesia

Anesthesia was used for CT and MRI examinations and also for coelioscopic validation of the reproductive organs. Hydration was ensured during anesthesia by using paper towels moistened with aged tap water (Supplementary Figure S2), as keeping the skin moist is critical for amphibians. For procedures that did not require long anesthesia (CT, coelioscopy), we anesthetized the frogs in a shallow water bath of 1 g/L tricaine-methanesulfonate (MS-222; Sigma-Aldrich, Steinheim, Germany) buffered to neutral pH with the same amount of disodium hydrogen phosphate. The animals were left in the bath for ca. 6 min, until their deep pain reflex was switched off, which we tested using tweezers by pinching the skin on their toes; if the animal did not pull the leg away, it was ready for the procedure. Using this method, anesthesia lasted ca. 40 min. For MRI examinations that required a longer time, the animals were anesthetized with an intramuscular injection of alfaxalone (20 mg/kg, Alfaxan^®^ Multidose 10 mg/ml; Zoetis Belgium SA, Rue Laid Burniat 1, 1348 Lovain-La-Neuve, Belgium). This method did not switch off the deep pain reflex but ensured immobility for ca. 100 min.

### 2.3 Ultrasound

On April 16 and 17, 2025, we performed preliminary ultrasound examinations on three immature individuals (one male, two females), and as a reference on a previously deceased gravid adult female stored in formalin. On May 20, seven further immature animals (two males, five females) were examined. Three out of the 10 frogs (one male and two females) were examined again on November 10, when the male had nuptial pads (i.e., reached sexual maturity) and one of the females was sexually mature whereas the other female was immature based on coelioscopic examination in October.

All ultrasonographic examinations were undertaken at the Csipet Csapat Veterinary Clinic, with real-time B mode ultrasound using Vetus 8 (Mindray, Shenzhen, China) ultrasound units, with a C11-3s Curved Array transducer nominal frequency range of 3.0−11.0 MHz. The images were taken with the following settings: other animal abdomen preset, Micro-convex Curved transducer Frequency range 4.7–12.8 MHz, Basic imaging mode with real-time images, Gain 55 dB, Frame Rate 30/s, Dynamic Range 115 dB, Depth 1.5–3.5 cm. The exact transmit frequency was not recorded. The ventral skin of the frog was moistened with aged tap water prior to ultrasonography for use as a conductor to enhance imaging. The ultrasound probe was used in the ventrodorsal directions. The ultrasonographic images of each frog were interpreted by up to three veterinarians whose primary practice focuses on treating dogs and cats, and whose ultrasound expertise is limited to general internal medicine and organ abnormalities in these two species. During the first ultrasonographic examinations, we also attempted to perform Doppler imaging, but only the dorsal aortae were detectable, so we did not systematically attempt it for every froglet.

### 2.4 Computed tomography

On May 24, we took eight immature animals (four males, four females) to Helvetic Dental Clinics. We used a human Cone Beam computed tomography (CBCT). Its large field of view (20 × 17 cm) and 450 μm resolution make it suitable for simultaneously imaging multiple experimental animals. We created a hand made three-level stand on which we placed the frogs to take CT scans of them (Supplementary Figure S3). Dental CBCT scan was performed using a Planmeca Viso G5 (Planmeca OY, Finland), and raw x-ray data were processed using Romexis 6.4.5.136 software. CBCT image stacks were analyzed using Romexis 6.4.5.136 (Planmeca OY). The CT images were taken with the following settings: FOV 20 × 10 cm, 450 μm resolution, 110 kV tube voltage/potential, 8.0 mA tube current, 5.625 s exposure time, 639 mGycm^2^ dose-area product (Supplementary Video S1).

### 2.5 Magnetic resonance imaging

On May 28, we examined four immature frogs (two males, two females) using MRI at the Quantitative *In vivo* Molecular Imaging Working Group at Semmelweis University, Institute of Biophysics and Radiation Biology. On October 7, we repeated the MRI examination on three out of the four frogs: the same individuals that were also re-examined by ultrasound in November. MRI was performed using a NanoScan PET/MRI scanner (Mediso Ltd., Hungary) having 3 T magnetic field, 600 mT/m gradient system and a volume transmit/receive coil with a diameter of 72 mm. Multiple sequence types were tested (Supplementary Table S1) to find the most appropriate ones, which provide the best contrast on the reproductive organs in reasonable acquisition time. The final protocol consisted of a T1-weighted spoiled gradient echo sequence with a three-dimensional acquisition scheme. The imaging parameters were as follows: 15 ms repetition time, 3.5 ms echo time, 40° flip angle, six averages, 46 × 30 × 27 mm^3^ field of view, 150 μm in plane resolution and 300 μm slice thickness in 17 min. Interview Fusion software (Mediso Ltd.) was used as the DICOM viewer software.

## 3 Results

Using ultrasonography, the eggs were clearly visible in the gravid female preserved in formalin (Figure 1). During the spring session, the sonographers could identify organs with lumen (intestines) or filled with air (lungs) and the beating heart, but parenchymal organs were not clearly distinguishable from each other in the small immature frogs. In the autumn, the veterinarians correctly recognized the female with mature eggs, while the ultrasonographic image of the female with undeveloped eggs did not clearly differ from that of the male with nuptial pads (Figure 1).

**Figure 1:**
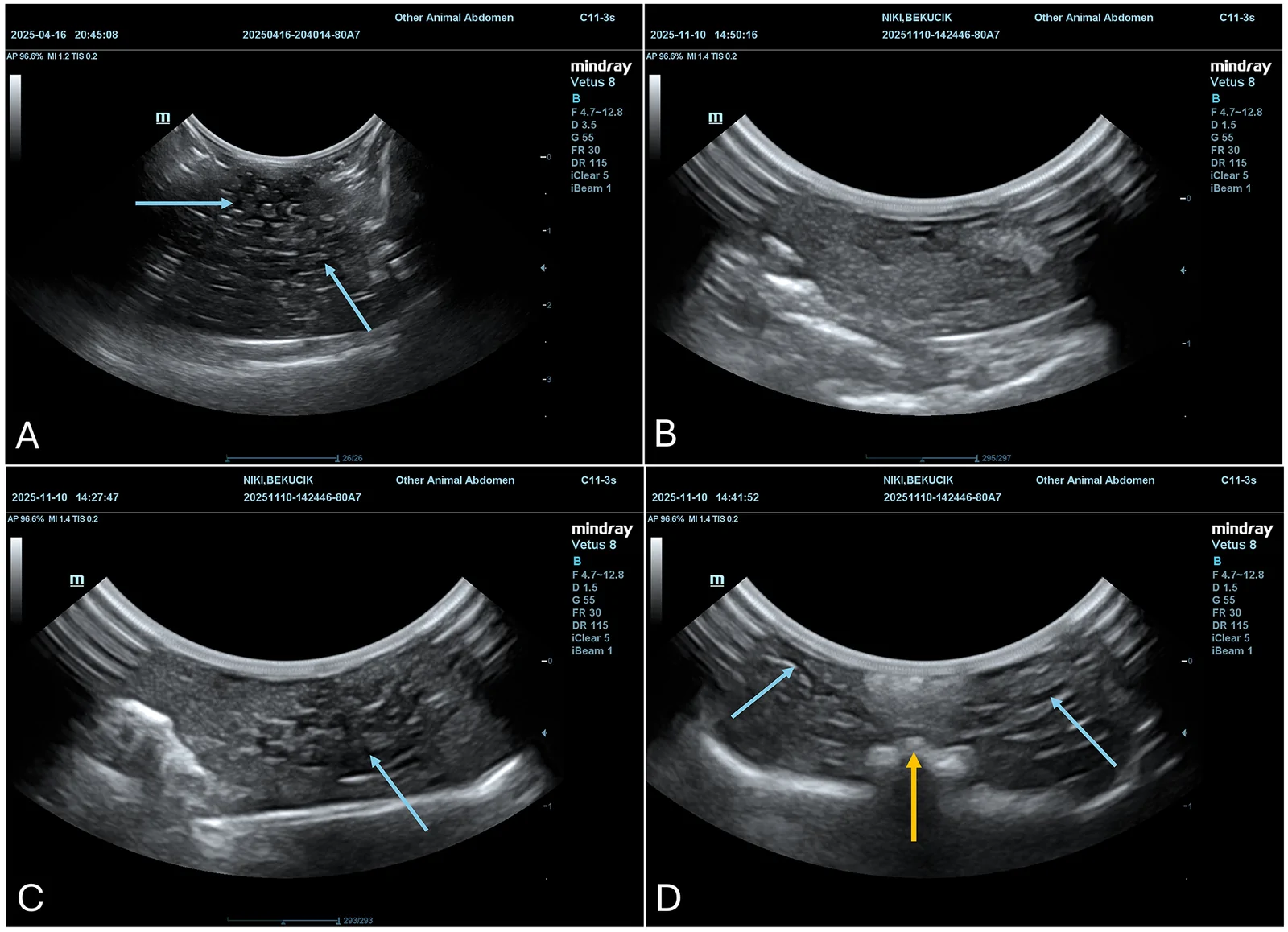
Panel **(A)** shows an ultrasound image of a gravid adult female with mature ova (the brighter, echodense areas among the darker, less echogenic areas, as indicated by the blue arrow), while Panel **(B)** shows an ultrasound image of a 2-year-old immature individual. In immature animals, most of the organs do not exhibit distinct echogenicity; therefore, the reproductive organs cannot be clearly distinguished from the internal organs. Panel **(C, D)** show ultrasound images of a 2.5-year-old female containing mature oocytes (lighter spots within dark, contiguous areas) in November, from two different viewing angles (Panel **C**) from the ventral surface toward the back; Panel **(D)** from the lower ventral surface toward the head; the orange arrow indicates the spine column).

Our handmade stand allowed simultaneous imaging of up to nine small animals using a CBCT device for human dentistry (Supplementary Figure S3, Supplementary Video S1). In the CT scans of immature frogs, bony and air-filled structures were recognizable, but no parenchymal organs were distinguishable (Supplementary Video S2). Therefore, we did not explore this method further with mature animals.

During MRI examination in the spring, when none of the individuals showed secondary sexual characteristics, the testes were already identifiable (Figure 2), but the ovaries were not yet clearly recognizable. In the autumn, phenotypic sex could be correctly determined in the male that already had nuptial pads and in the female whose ovaries were mature, but not in the other female whose ovaries were still immature (Figure 3). In addition, we were able to identify the following organs on the MRI images: the heart, liver, lungs, stomach, gallbladder, kidneys, muscles, vertebrae and spinal cord (Figures 2, 3).

**Figure 2:**
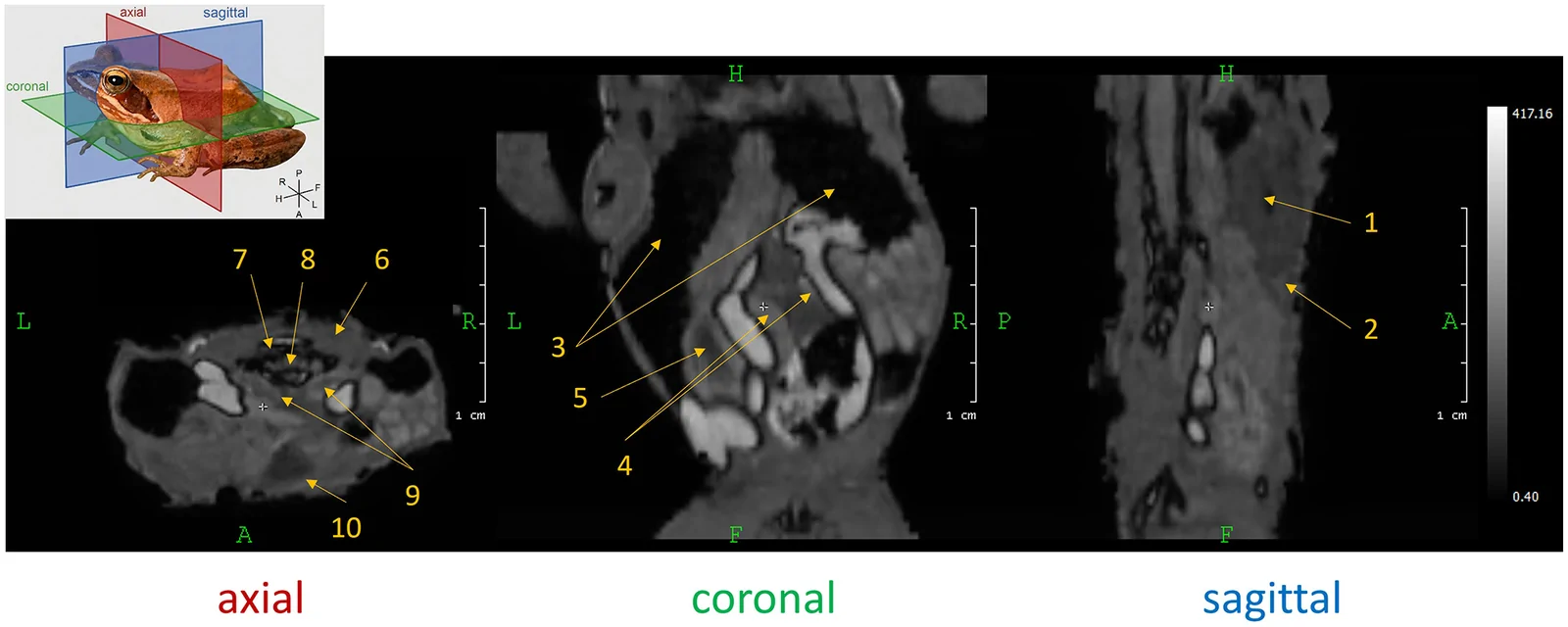
An MRI scan of a sexually immature 2-year-old male frog on three orthogonal cross-sectional views, where A, P, H, F, R, and L in the figure represent anterior (ventral), posterior (dorsal), head (cranial), feet (caudal), right, left, respectively. Each panel from left to right shows the same animal in axial/horizontal, sagittal, and coronal/vertical view, respectively. White crosshair marks the intersection of the three planes. Yellow arrows points to the organs: heart (1), liver (2), lungs (3), testes (4), stomach (5), muscle (6), vertebrae (7) spinal cord (8), kidneys (9) and gallbladder (10).

**Figure 3:**
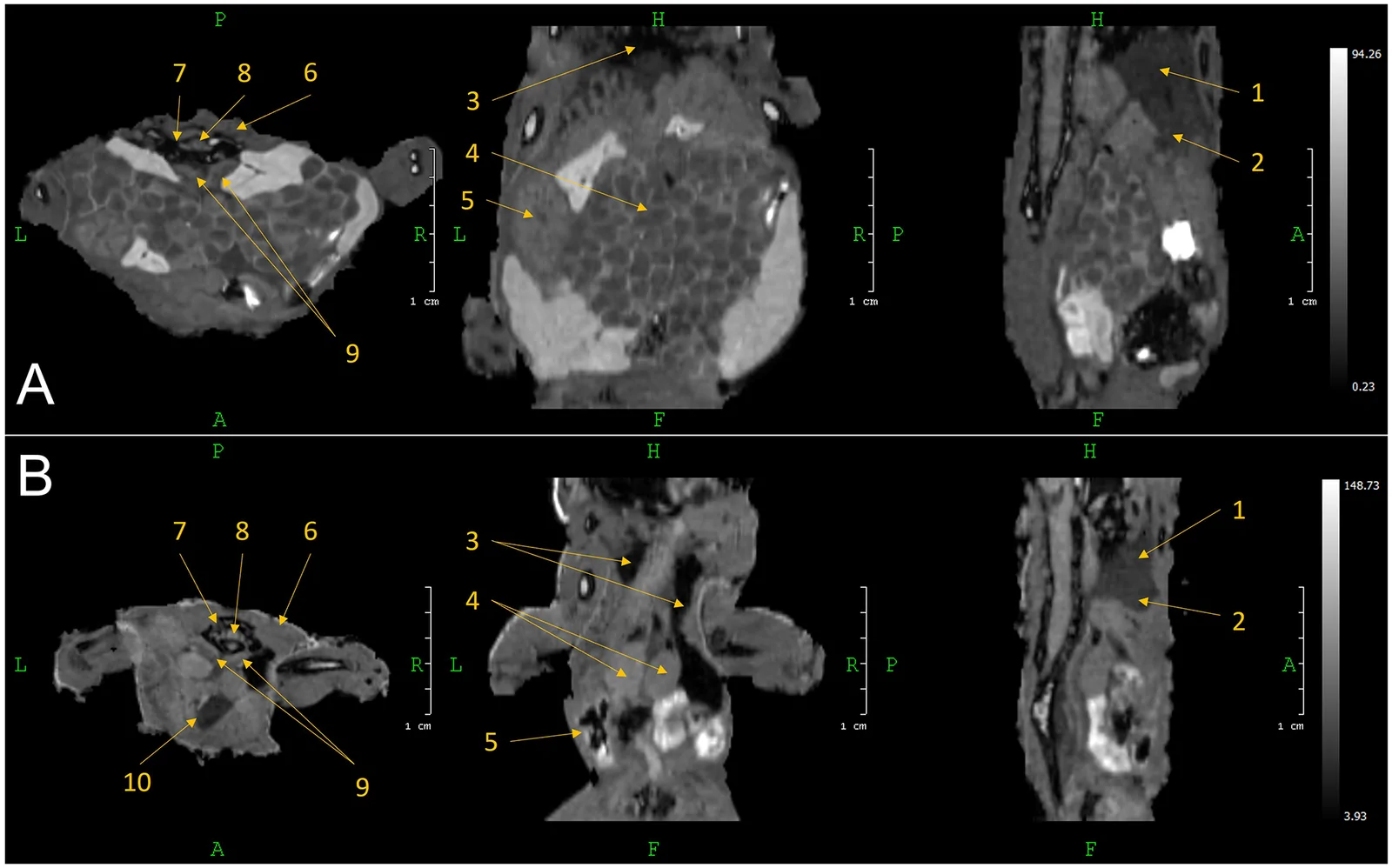
MRI images of 2.5-year-old frogs, showing a female filled with mature eggs **(A)** and a male presenting nuptial pads **(B)**, both shown on three orthogonal cross-sectional views (axial, coronal and sagittal from left to right respectively). Yellow arrows points to the organs: heart (1), liver (2), lungs (3), testes/ovary (4), stomach (5), muscle (6), vertebrae (7) spinal cord (8), kidneys (9) and gallbladder (10). See Figure 2 for further information about the interpretation of MRI images.

## 4 Discussion

In this study, we took the first step to investigate the feasibility of identifying the gonads of small, young agile frogs using non-invasive imaging techniques ranging from widely available ultrasonography to highly specialized CT and MRI scans. Each of these methods has been tested previously with adult mature animals in other amphibian species (37–39), and found suitable not only for sex determination but also for other reproduction-related studies such as assessing the state and fertility of the reproductive system (33, 40, 41). However, formal validation of these imaging techniques for sexing juveniles was missing. We found that none of the tested methods were suitable for fully reliable sex determination of both sexes in immature frogs, while they varied in usability for sexually mature animals.

Several articles describe the applicability of ultrasound examination in amphibians for medical purposes (28, 39, 42), for assessing reproductive status and for determining the sex of mature frogs (6, 16, 17, 27), with particular emphasis on identifying females. In these earlier studies, ultrasound examination was most suitable for identifying gravid females, as their ovaries are large and the easiest to see, whereas testes were rarely identifiable (6, 28, 39). Our study yielded similar results for agile frogs: mature ovaries were clearly visible, but we could not distinguish males and immature females. Previous studies that were successful in unambiguously recognizing testes were either done on ≥50 mm long animals (39) or used high-resolution ultrasound equipment with transducer frequency ranges of 17 and 22 MHz (16). In the latter study, the testes had a similar appearance to the kidneys, distinguished by their position, smaller size and less irregular edges (16). These subtle differences were identifiable by an experienced ultrasonographer only, pointing to the importance of practice when interpreting ultrasound images (16). In our study, the ultrasonographers used a device typically found in veterinary clinics, with a probe frequency range of 3.0–11.0 MHz. This device may have been insufficient to clearly visualize the nuanced differences between the testes and kidneys. In addition to this, our animals had a relatively small body size, and consequently smaller gonad size compared to the earlier studies that could identify mature males by ultrasound (16, 28). For example, while *Xenopus laevis* can range in size from 50 mm to as much as 140 mm, and male *Kaloula pulchra* from 54 to 70 mm, our largest specimen measured 50 mm. In both species, testicle size was at least 5 mm but could reach 10 mm [based on figures in (16)], whereas in our study on agile frogs, the testicles were ca. 2 mm at 2 years of age (Figure 2) and grew to ca. 4 mm by sexual maturity 6 months later (Figure 3). In *Ceratophrys cranwelli*, where the ovaries were easily identifiable by ultrasound while testes were correctly identified in 88.9% of cases, the visibility of the gonads increased linearly with body size (28). The smallest of the latter animals had an SVL of 53.4–58.8 mm, which is similar to the length of our largest specimens, further suggesting that small size may strongly constrain sex identification by ultrasound in anurans.

In the case of CT scans, earlier studies on amphibians were capable of visualizing internal organs, although the reproductive organs were rarely investigated. In 12–14 months old *Ceratophrys cranwelli* frogs, native (non-contrast) scans poorly differentiated coelomic organs due to low soft-tissue contrast, while contrast-enhanced angiography improved visualization of several structures including the ovaries, but still failed to clearly distinguish testes (28). Other authors used high-resolution micro-CT images of gravid *Eleutherodactylus atkinsi* females specifically to visualize the location of the mature eggs in the fallopian tube within the coelom cavity; however, this procedure is lethal (9). Despite using 450 μm resolution CBCT, we found that native 110 kV CT scans cannot be used in small-sized agile frogs for visualizing immature reproductive organs, due to relatively high tube voltage/potential compared to what would be ideal for visualization of contrast between soft tissues. Contrast-enhanced CT scans can provide better results for visualizing various soft tissues and organs or even lymphatic flow in amphibians (28, 40), and these articles did not report any side effects or deaths. However, we were reluctant to apply contrast media to our frogs because they were part of a long-term experiment. The potential side effects of contrast agents in amphibians are not known at all, but since these animals are generally sensitive to chemical substances (43), this would require further investigation. Studies attempting sexing by CT with and without contrast and tracking the health of the animals for a long follow-up period would be useful (16).

MRI has been found successful for identifying the gonads and gravidity in other amphibian species (*Xenopus laevis* and *Kaloula pulchra*), although in both species the specimens examined were mature adults with relatively large body sizes (16). Our results show that MRI is also successful in sexing mature agile frogs with a relatively small body size, but not for immatures, as only the testes were recognizable in juvenile animals with the scanning parameters we explored (Supplementary Table S1). In order to achieve adequate MRI image contrast, it is essential to test several sequence types in advance, preferably on individuals of known sex, to find the most suitable parameter settings. Three-dimensional imaging schemes proved to be the most informative; although it is time-consuming, it produces isovoxel images that allow for orientation in all three directions (axial, sagittal, coronal) (44). However, the scanning time is limited by the duration of anesthesia in amphibians, which increases the risk of complications and even mortality. Furthermore, MRI examinations might cause damage to or interfere with the hearing of experimental animals (45), although this has not yet been tested in amphibians, even though mating calls play an important role in reproduction within this species group (46). For these reasons, gonad identification using MRI scans may not be the best solution in terms of animal welfare, especially if the animals are to be raised further or released back into their environment. Furthermore, the procedure is costly and not widely available. These drawbacks limit the usability of MRI for sexing amphibians.

Taken together, identifying phenotypic sex in small-sized juvenile frogs with non-invasive imaging methods remains an unresolved challenge. Although immature testes are detectable with MRI, we could not unambiguously recognize immature ovaries with any of the imaging techniques used in our study. However, when female frogs begin to produce mature eggs, despite their small size, both small-animal translational field strength MRI and the more widely available modality of ultrasonography can reliably reveal mature ovaries. Ultrasound imaging is also faster and involves less risk, as it can be performed without sedating the animals (6, 27), and diagnostic ultrasonography using 4.7–12.8 MHz transducers is available in many veterinary clinics. Therefore, it would be worthwhile to further investigate the utility of proper high-frequency ultrasound techniques for sexing juvenile amphibians, particularly the high-resolution ultrasound that was used successfully in large, mature frogs of both sexes in an earlier study (16).

## Data Availability

The original contributions presented in the study are included in the article/Supplementary material, further inquiries can be directed to the corresponding authors.
